# Sport, Refugees, and Forced Migration: A Critical Review of the Literature

**DOI:** 10.3389/fspor.2019.00047

**Published:** 2019-10-11

**Authors:** Ramón Spaaij, Jora Broerse, Sarah Oxford, Carla Luguetti, Fiona McLachlan, Brent McDonald, Bojana Klepac, Lisa Lymbery, Jeffrey Bishara, Aurélie Pankowiak

**Affiliations:** ^1^Institute for Health and Sport, Victoria University, Melbourne, VIC, Australia; ^2^Department of Sociology, University of Amsterdam, Amsterdam, Netherlands

**Keywords:** forced migration, refugee, sport participation, physical activity, integration, health promotion, leisure, asylum seeker

## Abstract

Researchers, policy makers, and practitioners increasingly pay attention to sport and physical activity as a means and context for refugee wellbeing and integration, influenced by wider political and policy concerns about forced migration. Considering this growing scholarly and policy attention, it is timely to take stock of, and critically reflect on, recent developments in this field of research. This paper offers an integrative, critical review of the scientific literature on the topic. It critically synthesizes what is known about the sport and physical activity experiences of people with refugee and forced migrant backgrounds, and identifies key issues and directions for future research in this field. This review of contemporary academic literature comprises 83 publications derived from fourteen languages published between 1996 and 2019. It shows a substantial increase in the volume of published research on the topic in recent years (2017–2019). Published research is concentrated primarily in Western countries around the themes of health promotion, integration and social inclusion, and barriers and facilitators to participation in sport and physical activity. The findings foreground the use of policy categories, deficit approaches, and intersectionalities as three pressing challenges in this area of research. Based on this synthesis, the authors identify four research gaps that require attention in future research: the experiential (embodied emotional) dimensions of sport and physical activity, the need to decolonize research, the space for innovative methodologies, and research ethics.

## Introduction

The field of refugee and forced migration studies has grown considerably over the last 60 years (Black, 2001; Neumann et al., 2014). Additionally, there is a vast scholarly literature on migration that does not explicitly self-identify as “refugee or forced migration studies,” but which still deals substantively with refugees and forced migrants as its subject (Black, 2001). The existing scholarship on refugees and forced migrants covers a broad spectrum of issues, experiences and impacts, and has been closely connected to political and policy developments. Sport and physical activity have historically received scant attention within the field of refugee and forced migration studies. For example, a mere 10 of the 1,451 publications listed in Neumann's (2016) authoritative bibliography on the settlement of refugees in Australia (1940–2016) address sport and physical activity. This relative lack of sports-focused refugee and forced migration studies is somewhat surprising considering the rich tradition of research on the nexus between sport and transnational migration more broadly (Carter, 2011; Maguire, 2013; Agergaard, 2018).

In recent years, in parallel with political and policy concerns about forced migration, the attention for sport as a means or context for refugee wellbeing and settlement has been on the increase in research, policy and practice (Spaaij and Oxford, 2018). The European Union (EU), North America, and Australia have witnessed significant investment in sports programs and interventions that aim to engage refugees and asylum seekers in sport and physical activity for health, therapeutic, or social purposes. Between 2016 and 2018 alone, the European Commission awarded a total of €3 million across 54 projects that aimed to support the opportunities that sport and physical activity provide to promote social inclusion and wellbeing of refugees in EU member states (European Commission, 2018). In addition, the EU's Erasmus+ program co-funded two international projects—Activity, Sport and Play for the Inclusion of Refugees in Europe (ASPIRE) and Integration of Refugees through Sport (IRTS)—which bring together research and practice in order to enhance sport organizations' capacity to deliver suitable participation opportunities for migrants and refugees. It has also co-funded the Sport Inclusion Network (SPIN) and its successor, the European Sport Inclusion Network (ESPIN), which promotes the civic participation of migrants through sport volunteering. Similar, albeit mostly smaller-scale, projects have been undertaken in countries, such as Canada and Australia.

Given the scholarly and policy attention afforded to sport and physical activity as a means or context for promoting refugee wellbeing and integration, it is timely to take stock of, and critically reflect on, recent developments in this field of research. No integrative, critical reviews of the current state of knowledge in this field have been produced to date, and very few studies have engaged with non-English language publications on the topic. The purpose of this paper is to critically synthesize what is known about the sport and physical activity experiences of people with refugee and forced migrant backgrounds, and to identify promising directions for future research in this field. Key terms, such as “refugee” and “forced migrant,” are unpacked and discussed throughout the paper. We critically survey the scholarly literature in order to identify the key concepts, themes, debates, and challenges, as well as knowledge gaps and unanswered questions that warrant further research. Critical, in-depth synthesis of the current state of play in this field of research can not only advance theoretical and methodological understandings, but also inform policy and practice aimed at enhancing refugees and forced migrants' access to sport and physical activity and associated health and social outcomes. The evidence and insights presented can be used to inform existing and future sports-based programs and interventions in order to strengthen their conceptual foundation, design, processes, equity, and impact.

This paper is structured as follows. The next section describes the methodology deployed in this review of the literature. This is followed by a discussion of key concepts, issues, themes, and problems. Then, we turn to current research gaps and identify future developments and promising directions for this field of research.

## Methods

This critical narrative review seeks to provide a comprehensive assessment and critical reading of the scholarly literature on sport and physical activity experiences of people with refugee and forced migrant backgrounds. A narrative review approach is well suited for evaluating this emerging and evolving field of research (Bryman, 2015). The study of sport, refugees, and forced migration is characterized by fluid and evolving boundaries (e.g., regarding what constitutes “forced”) and low consensus concerning key research questions and theoretical approaches. The review includes studies of any design that investigated the engagement of refugees and forced migrants in sport and physical activity, either as participants, volunteers, or fans. Journal articles, books, book chapters, peer-reviewed research reports, and published PhD and Master's theses were all included in the review. For an emergent area of study, such as this, it is essential to consider publication types, such as PhD and Master's theses that may take some time to be published in peer-reviewed scientific journals. We included both empirical studies and theoretical publications. All non-peer-reviewed publications were excluded. Thus, conference abstracts, working papers, non-refereed reports, and other gray literature were excluded.

The literature search covered fourteen languages: English, Croatian, Dutch, Farsi, French, German, Indonesian, Italian, Japanese, Portuguese, Slovenian, Spanish, Swedish, and Turkish. This multi-language literature review process was enabled by the linguistic diversity of the research team. The authors include English, Dutch, Portuguese, and Croatian native speakers and they master several other languages (French, German, Italian, Japanese, Slovenian, and Spanish). The authors received research assistance from colleagues and postgraduate students whose native languages are Indonesian, Turkish, Farsi (Persian), and Swedish. In doing so, this review deliberately seeks to move beyond an exclusively Anglo-centric focus to incorporate studies conducted in other parts of the world that can inform theoretical and practical understandings of the relationship between sport and forced migration. Relevant publications were identified for eleven of the fourteen languages.

Publications were identified using seven electronic databases: Scopus, EBSCOhost, Web of Science, J-STAGE, CiNii, Open Access Theses and Dissertations (OATD), and Google Scholar. The keywords “sport,” “physical activity,” “exercise,” “movement therapy,” and “leisure” were combined with refugee descriptor keywords “refugee,” “asylum seeker,” “forced migrant,” and “forced migration” to gain a comprehensive coverage of the scholarly literature. Linguistically and culturally appropriate translations of these search terms were used for foreign-language publication searches. In addition, we searched the reference lists and bibliographies of publications for further relevant references that we followed up (Bryman, 2015). Existing bibliographies, such as the ASPIRE literature review (Bailey et al., 2017) and Bertram's et al. (2016) *Mapping of good practices relating to social inclusion of migrants through sport* were also scrutinized for relevant references. Furthermore, we personally contacted six researchers from other countries to request that they identify any additional publications in their native languages that they deemed relevant to the purpose of this review. This process resulted in the inclusion of an additional four foreign-language publications. Finally, one additional publication was identified by the reviewers during the peer review process for this paper.

There was no time restriction on the publications. The oldest publication identified during the search was Russell and Stage's (1996) study of the meaning of leisure among Sudanese women in a refugee camp in Kakuma, Kenya. We continued the literature search until we reached saturation point, where we saw the same citations repeated on a regular basis and no new citations were generated. Journal articles that were available online but not yet published in print were also included.

The literature search resulted in a total of 83 publications that were included in the review. This total is unlikely to be exhaustive, however due to the comprehensive literature identification process it represents the vast majority of scientific research on the topic to date (by 30 June 2019, the cut-off point for the literature search). Table 1 summarizes basic information and contexts of the studies included in the review. Fifty-eight (71%) publications comprised peer-reviewed journal articles. The remaining publications included eight Master's theses (10%), five PhD theses (6%), four book chapters (5%), four research reports (5%), and two Bachelor theses (2%). The final sample of publications covered eleven languages: English (68 publications, or 83%), Turkish (three), German (two), Dutch (two), Spanish (one), Portuguese (one), Italian (one), Japanese (one), Croatian (one), Slovenian (one), and Indonesian (one). Only a handful of authors published multiple studies (single authored or with colleagues) that were included in the review: Ramón Spaaij, Clemens Ley, Enrico Michelini, Meredith Whitley, and Chris Stone (see Table 1).

**Table 1:** Publications included in the review.

**Year**	**Author**	**Title**	**Country (research site)**	**Type**
1996	Russell and Stage	Leisure as burden: Sudanese women in refugee camps	Kenya	Journal article
1999	Kruiswijk	Vrijetijdsbesteding van jonge vluchtelingen [Leisure activities of young refugees]	Netherlands	Research report
2003	Guerin et al.	Physical activity programs for refugee Somali women: Working out in a new country	New Zealand	Journal article
2003	Voskanyan et al.	Life and leisure among young adult war refugees in the South Caucasus	Georgia, Armenia	Journal article
2004	Amara et al.	The roles of sport and education in the social inclusion of asylum seekers and refugees	United Kingdom	Journal article
2006	Ogino Takahiro	わが国における難民受入れと公的支援の 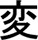 遷[Transition of refugee acceptance and public support by Japan]	Japan	Journal article
2006	Thachuk	Sport for refugee children	Not applicable	Master's thesis
2007	Harris	Dance/movement therapy approaches to fostering resilience and recovery among African adolescent torture survivors	United States, Sierra Leone	Journal article
2008	Olliff	Playing for the future: The role of sport and recreation in supporting refugee young people to “settle well” in Australia	Australia	Journal article
2009	Hancock, Cooper, and Bahn	Evaluation of a youth CaLD (cultural and linguistically diverse) sports program in Western Australia: Resettling refugees using sport as a conduit to integration	Australia	Journal article
2009	Henry	Estrategias para la integración social a través del deporte: el uso del deporte para la integración social entre refugiados y solicitantes de asilo [Strategies for social integration through sport: The use of sport for the social integration of refugees and asylum seekers]	United Kingdom	Book chapter
2009	Hertting and Karlefors	Sport as a context for integration: Newly arrived immigrant children in Sweden drawing sporting experiences	Sweden	Journal article
2009	Meier	Zum ersten Mal im Leben umarmt: Sport und Spiel als Mehrwert für Kinderflüchtlinge [Embraced for the first time in life: The added value of sport and play for refugee children]	Germany	Book chapter
2009	Northcote and Casimiro	A critical approach to evidence-based resettlement policy: Lessons learned from an Australian Muslim refugee sports program	Australia	Journal article
2009	Palmer	Soccer and the politics of identity for young Muslim refugee women in South Australia	Australia	Journal article
2009	Stack and Iwasaki	The role of leisure pursuits in adaptation processes among Afghan refugees who have immigrated to Winnipeg, Canada	Canada	Journal article
2009	Wright	Understanding the role of sport for development in community capacity building in a refugee camp in Tanzania	Tanzania	Master's thesis
2010	Evers	Intimacy, sport and young refugee men	Australia	Journal article
2010	Nathan et al.	Social cohesion through football: a quasi-experimental mixed methods design to evaluate a complex health promotion program	Australia	Journal article
2010	Whitley and Gould	Psychosocial Development in Refugee Children and Youth through the Personal–Social Responsibility Model	United States	Journal article
2011	Belore	“Young women growing graciously”: Considering sport, gender and development in diasporic space	Canada	Master's thesis
2011	Hashimoto-Govindasamy, and Rose	An ethnographic process evaluation of a community support program with Sudanese refugee women in western Sydney	Australia	Journal article
2011	Huffman	Using Sport to Build Community: Service Learning with Iraqi Refugees	United States	PhD thesis
2011	Osazee	Liminal Belonging: West African male asylum seekers' narratives of the asylum experience whilst in Finland	Finland	Master's thesis
2012	Renzaho, McCabe, and Swinburn	Intergenerational differences in food, physical activity, and body size perceptions among African migrants	Australia	Journal article
2012	Spaaij	Beyond the playing field: Experiences of sport, social capital and integration among Somalis in Australia	Australia	Journal article
2012	Wieland et al.	Physical activity and nutrition among immigrant and refugee women: A community-based participatory research approach	United States	Journal article
2013	Bunde-Birouste	Kicking goals for social change: An autoethnographic study exploring the feasibility of developing a program that harnesses the passion for the World Game to help refugee youth settle into their new country	Australia	PhD thesis
2013	Ha and Lyras	Sport for refugee youth in a new society: The role of acculturation in sport for development and peace programming	Not applicable	Journal article
2013	Nathan et al.	“We wouldn't of made friends if we didn't come to Football United”: The impacts of a football program on young people's peer, prosocial and cross-cultural relationships	Australia	Journal article
2013	Spaaij	Cultural diversity in community sport: An ethnographic inquiry of Somali Australians' experiences	Australia	Journal article
2013	Stone	Football: A shared sense of belonging?	United Kingdom	Research report
2013	Uptin, Wright, and Harwood	“It felt like I was a black dot on white paper”: Examining young former refugees' experience of entering Australian high schools	Australia	Journal article
2014	Juhart and Cof	Beg mišic: Migracije v športu ali šport kot nova migracijska strategija [Muscle drain: Migration in sport or sport as a new migration strategy]	Slovenia	Research report
2014	Mohamed et al.	Physical activity among Somali men in Minnesota: Barriers, facilitators, and recommendations	United States	Journal article
2015	Jeanes, O'Connor, and Alfrey	Sport and the resettlement of young people from refugee backgrounds in Australia	Australia	Journal article
2015	Nordbrandt et al.	Treatment of traumatized refugees	Denmark	Journal article
2015	Solling	Football as a tool for integration	Sweden	Bachelor thesis
2015	Spaaij	Refugee youth, belonging and community sport	Australia	Journal article
2016	O'Driscoll	Exploring cultural variables affecting sport and physical activity behaviors of Karen refugees in Australia: Applying a culturally specific approach to active lifestyles	Australia	PhD thesis
2016	Pizzolati and Sterchele	Mixed-sex in sport for development: A pragmatic and symbolic device	Italy	Journal article
2016	Rosso and McGrath	Promoting physical activity among children and youth in disadvantaged South Australian CALD communities through alternative community sport opportunities	Australia	Journal article
2016	Whitley, Coble, and Jewell	Evaluation of a sport-based youth development programme for refugees	United States	Journal article
2017	Baker-Lewton, Curnow, and Sonn	The community soccer club: A story to tell. Building an intercultural soccer hub in Melbourne's West	Australia	Research report
2017	Baker-Lewton et al.	“I haven't lost hope of reaching out…”: exposing racism in sport by elevating counternarratives	Australia	Journal article
2017	Bartsen	De inzet van sport als middel bij Syrische vluchtelingen [Sport as means for Syrian refugees]	Netherlands	Bachelor's thesis
2017	Block and Gibbs	Promoting social inclusion through sport for refugee-background youth in Australia: Analyzing different participation models	Australia	Journal article
2017	Dukic, McDonald, and Spaaij	Being able to play: Experiences of social inclusion and exclusion within a football team of people seeking asylum	Australia	Journal article
2017	Hartley, Fleay, and Tye	Exploring physical activity engagement and barriers for asylum seekers in Australia coping with prolonged uncertainty and no right to work	Australia	Journal article
2017	Hurly and Walker	“When you see nature, nature give you something inside”: The role of nature-based leisure in fostering refugee well-being in Canada	Canada	Journal article
2017	Kallas	Understanding the perspectives of Syrian refugee women toward their health and physical activity needs as they become integrated into Canadian society	Canada	Master's thesis
2017	Özsari, Fişekçioğlu, and Altin	Gençlik merkezi faaliyetlerine katılan Suriyeli mültecilerin algıladıkları hizmet kalitesi üzerine bir araştırma [A study of the quality of service perceived by Syrian refugees participating in youth center activities]	Turkey	Journal article
2017	Ley et al.	Exploring flow in sport and exercise therapy with war and torture survivors	Austria	Journal article
2017	Trigo and Freitas	O futebol como instrument politico na crise migratória na Alemanha e na Europa [Football as political instrument in the immigration crisis in Germany and Europe]	Germany	Journal article
2017	Woodhouse and Conricode	In-ger-land, In-ger-land, In-ger-land! Exploring the impact of soccer on the sense of belonging of those seeking asylum in the UK	United Kingdom	Journal article
2018	Abur	Settlement Strategies for the South Sudanese Community in Melbourne: An Analysis of Employment and Sport Participation	Australia	PhD thesis
2018	Apriadi and Yuliantoro	Perlindungan hak asasi manusia pengungsi lintas batas di Rumah Detensi Imigrasi (Rudenim) Indonesia (studi case: Rudenim Surabaya) [The protection of the human rights of crossborder refugees in Indonesian immigration detention facilities (case study: Rudenim Surabaya)]	Indonesia	Journal article
2018	Atali	Türkiye'de Suriyelilere saǧlanan hizmetlerin spor yönü ile incelenmesi [An analysis on the services provided to the Syrians in Turkey in terms of sport]	Turkey	Journal article
2018	Blanchard	More than a game? Exploring sport's role in refugee and asylum-seeker settlement in Glashow, Scotland	United Kingdom	Master's thesis
2018	Burrmann et al.	Sport offers for refugees in Germany: Promoting and hindering conditions in voluntary sport clubs	Germany	Journal article
2018	Doidge	Refugees united: The role of activism and football in supporting refugees	France	Book chapter
2018	Fader	Bonding over the love of soccer is no joke: A mixed method study exploring sense of community, resilience, and cultural adjustment for refugee youth participants	United States	Master's thesis
2018	Kavi and Yildirim	Çocuk ve gençlik politikalari açisindan sporun önemi ve Suriyeli mültecilere yönelik uygulamal [The importance of sport in terms of children and youth policing and the increase of Syrian refugees]	Turkey	Journal article
2018	Ley, Rato Barrio, and Koch	“In the sport I am here”: Therapeutic processes and health effects of sport and exercise on PTSD	Austria	Journal article
2018	McGee and Pelham	Politics at play: locating human rights, refugees and grassroots humanitarianism in the Calais Jungle	France	Journal article
2018	Michelini	War, migration, resettlement and sport socialization of young athletes: The case of Syrian elite water polo	Europe (mainly Sweden, Germany, Netherlands)	Journal article
2018	Ravaglia	L'Inclusione sociale dei richiedenti asilo tramite lo sport: l'esempio della Polisportiva San Precario [Social inclusion of asylum seekers through sport: The case of Polisportiva San Precario]	Italy	Master's thesis
2018	Spaaij and Broerse	Sport and the politics of belonging: The experiences of Australian and Dutch Somalis	Australia, Netherlands	Book chapter
2018	Stone	Utopian community football? Sport, hope and belongingness in the lives of refugees and asylum seekers	United Kingdom	Journal article
2018	Waardenburg et al.	Sport in liminal spaces: The meaning of sport activities for refugees living in a reception center	Netherlands	Journal article
2018	Seiberth, Thiel, and Hanke	Flüchtlinge als neue Zielgruppe des organisierten Sports. Eine Pilot-Studie zur Entwicklung von Integrations-projekten für Geflüchtete in Sportvereinen	Germany	Journal article
2019	Anderson et al.	Managerial perceptions of factors affecting the design and delivery of sport for health programs for refugee populations	Germany, Netherlands	Journal article
2019	Bangura	Exploring a sense of belonging amongst African refugees in the North of England: What influences participation and engagement	United Kingdom	Master's thesis
2019	Capalbo	The role of a soccer-based program in the acculturation of refugee youth: A retrospective examination	United States	PhD thesis
2019	Hurly	“I feel something is still missing”: Leisure meanings of African refugee women in Canada	Canada	Journal article
2019	Jurkovic	Migranti i sport: Nogomet kao prostor integracije izbjeglica u Hrvatskoj [Migrants and sport: Football as an area for integration of refugees in Croatia]	Croatia	Journal article
2019	Ley and Rato Barrio	Promoting health of refugees in and through sport and physical activity: a psychosocial, trauma-sensitive approach	Not applicable	Book chapter
2019	McDonald, Spaaij, and Dukic	Moments of social inclusion: Asylum seekers, football and solidarity	Australia	Journal article
2019	Mohammadi	Social inclusion of newly arrived female asylum seekers and refugees through a community sport initiative: The case of Bike Bridge	Germany	Journal article
2019	Robinson et al.	The Syrian Canadian Sports Club: A community-based Participatory Action Research project with/for Syrian youth refugees	Canada	Journal article
2019	Spaaij and Broerse	Diaspora as aesthetic formation: Community sports events and the making of a Somali diaspora	Netherlands	Journal article
2019	Stura	“What makes us strong”: the role of sports clubs in facilitating integration of refugees	Germany	Journal article

### Data Analysis

The publications were analyzed systematically by inputting data from the literature into a spreadsheet matrix. The matrix included 21 variables: author, year of publication, title, country, cultural group, mode of identification, refugee descriptor, geographical setting, type of research, methodology, sample, theory used, type of sport/physical activity, organizational form, main themes, main findings, main conclusions, limitations, recommendations for research, recommendations for policy/practice, and ethical considerations. A codebook was developed that provided a descriptor and response categories for each variable. This matrix helped to increase consistency, transparency and inter-coder reliability in the data analysis, allowing for systematic comparison across the studies and ensuring that the data collected from the publications would be meaningful to the purpose of the review. Dialogue among the investigators resulted in intersubjective agreement on coding decisions. All observations regarding the quality of particular aspects of the studies, such as theory, methodology, and ethics were documented in the matrix. In the remainder of this paper, we present the findings of this review process.

## Evolution of the Literature

**Figure 1:**
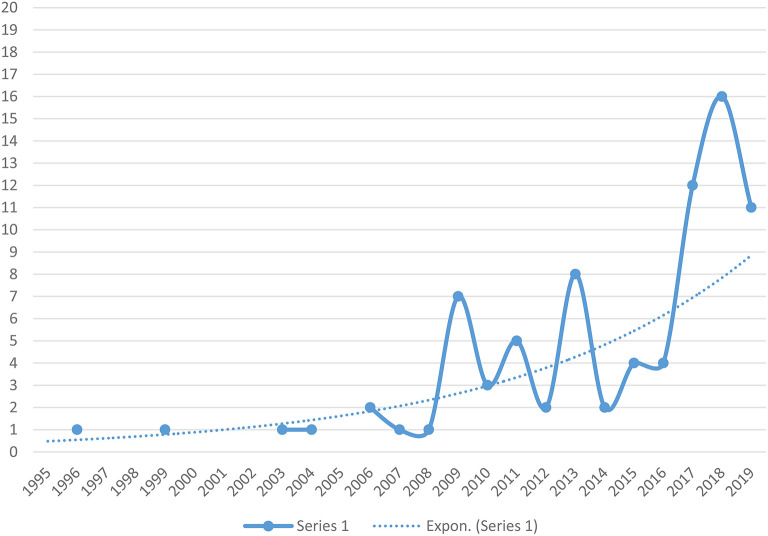
Evolution of scientific literature on sport and refugees, 1995–2019.

In total, 83 publications were included in this literature review. As Figure 1 shows, the volume of published research on the topic has been on the increase in recent years, most notably from 2017 onwards. Although it is too early to determine whether this will constitute a longer-term trend, the trend line does seem to reflect the aforementioned increase in political and public attention for the role of sport and physical activity in the settlement and wellbeing of refugees and forced migrants in Western countries. The historical development of scholarship in this field should be viewed in the context of its low base rate vis-à-vis other areas of refugee and forced migration research.

The evolution of the literature can be broken down into three phases. The first phase comprises the period up to 2008, during which scant academic attention was paid to the sport and physical activity experiences of people with refugee and forced migrant backgrounds. Between 1995 and 2008, the number of publications directly addressing the topic was minimal, at an average of 0.57 published studies per year (eight publications in total, or 10% of the retrieved literature). The two most cited publications that appeared during this period are Amara's et al. (2004) report on the roles of sport and education in the social inclusion of asylum seekers and refugees in the United Kingdom, and Olliff's (2008) applied study of sport and recreation in the settlement of refugee-background young people in Australia. Two caveats apply to this observation. First, it is possible that some research on sport and migration more broadly covered aspects of refugee experience of sport and physical activity, without using the terms “refugees” or “forced migrants” explicitly (i.e., it focused on migrants more broadly which may have included forced migrants, for example as research participants). Second, we recognize that not all of the publications produced during the earlier period are available in electronic format and retrievable through electronic searches. It is possible that some older publications were not detected through our literature search processes.

The second phase extends from 2009 to 2016 and is characterized by a relatively steady flurry of published research on the topic. Thirty-five publications (42%) in our review were published during this period, at an average of four publications per year. The third phase, from 2017 onwards, features a considerable acceleration of the publication rate. The years 2017 and 2018 had twelve and sixteen publications, respectively, while the first half of 2019 (up to 30 June) already produced eleven publications. The total number of publications in this two-and-a-half-year period was 39, which corresponds to nearly half (46%) of the entire sample.

## Characteristics of the Studies

### Geographical Locations

**Figure 2:**
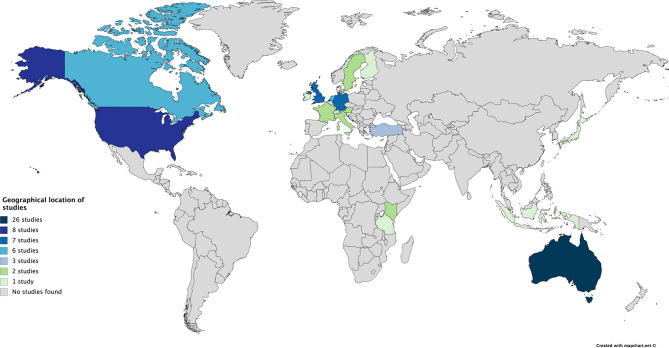
Geographical location of studies.

Figure 2 visualizes the countries where the empirical data reported in the publications were collected. These countries thus represent the research sites, and not necessarily the authors' countries of origin. While the geographical locations of the literature are diverse, a clear pattern can be identified in that the vast majority of studies (*n* = 75, or 89%) were undertaken in (re)settlement countries. The country from which most of the published research originates is Australia, which produced more than one-third of the publications included in the review (*n* = 26, or 31%). Forty percent of the studies were based in Europe (*n* = 33), most notably Germany (9%), United Kingdom (9%) and the Netherlands (7%); the remaining European studies were conducted in a variety of EU member states. The United States (10%) and Canada (7%) were also strongly represented in the sample. Three publications (4%) came from Turkey; all of which focused on Syrian refugees in the country. Only five studies (6%) were situated in the Global South, namely in Kenya (two), Tanzania, Sierra Leone, and Indonesia. Some studies were based in multiple geographical locations, notably those that involved comparative analysis (e.g., Harris, 2007; Spaaij and Broerse, 2018). In four non-empirical publications the country was not applicable.

The geographical distribution of the academic literature appears to reflect the Eurocentric bias of global knowledge production in the social and health sciences (Connell, 2007; Keim et al., 2014) and in sports studies (Spaaij et al., 2014). Of particular note is that there is a discrepancy between the research sites of the published studies and the locations where the majority of the world's displaced people actually reside. In 2017, developing regions hosted 85 per cent of the world's refugees under the United Nations High Commissioner for Refugees' (UNHCR) mandate, about 16.9 million people (UNHCR, 2018). The least developed countries provided asylum to one-third of the global total (6.7 million refugees). The main countries of asylum for refugees in 2017 were Turkey, Pakistan, Uganda, Lebanon, Islamic Republic of Iran, Germany, Bangladesh, and Sudan (UNHCR, 2018). Turkey and Germany are the only two countries on this list that were represented as research sites in the literature included in this review. Moreover, it is noteworthy that very few authors themselves have refugee or forced migrant backgrounds. Only three authors of literature covered in this review explicitly self-identified as having a refugee background: Darko Dukic (in Dukic et al., 2017; McDonald et al., 2019), William Abur (Abur, 2018), and David Nyuol Vincent (in Baker-Lewton et al., 2017). The publications of these three authors were affiliated with Victoria University in Melbourne, Australia. In addition, two authors explicitly disclosed their own (voluntary) migration experiences (Spaaij, 2013; Mohammadi, 2019). These publications inform our understanding of insider/outsider perspectives on the topic and sensitize us toward the importance of explicit engagement and reflection on issues of positionality and the politics of representation in research, such as the extent to which research is conducted *on* or *for*, rather than *with* or *by*, refugees and forced migrants.

### Types of Sport and Physical Activity

The literature search terms were deliberately inclusive of a wide range of sport and physical activities, which is reflected in the identified publications. The publications indicate the diverse forms of sport and physical activity in which refugees and forced migrants (aspire to) participate. The main loci of study in the literature are sports and physical activity interventions, and dance/movement therapy programs (41 publications, or 49%). These interventions were located across a variety of settings including community health facilities, settlement programs, participatory action projects, refugee camps, and immigration detention centers. Within the interventions, sport and physical activity were primarily used as “plus sport” (Coalter, 2007). These interventions gave primacy to social, health, and educational objectives beyond sport participation itself; that is, they used sport and physical activity as part of a broader and more complex set of processes designed to engage, “empower,” or assimilate refugees and forced migrants.

Twenty-seven publications (33%) focused on organized team sports (i.e., voluntary sports clubs, school-based competitions), such as football (soccer), basketball, cricket, volleyball, ultimate frisbee, netball, and Australian football, with football the dominant sport being studied. Ten publications (12%) examined active recreation or informal physical activity, such as fitness exercise, swimming, running, and badminton. Thus, despite the growing prominence of informal sport as a site for physical activity (Jeanes et al., 2019), relatively few studies examine refugee and forced migrants' involvement in informal sport and physical activity. Finally, two studies (2%) focused exclusively on *ad-hoc*, community-driven sports events, such as tournaments and festivals (Osazee, 2011; Spaaij and Broerse, 2019); while others examined a mix of *ad-hoc* events and programs/clubs (e.g., Spaaij and Broerse, 2018; Stone, 2018).

All but one publication focus primarily on non-professional sports activities. The main exception is Michelini (2018), who explores the elite sport experiences of forced migrants by describing Syrian elite water polo players' socialization journeys into their new countries. Michelini (2018: p. 17) concludes that participation in elite-level water polo supports settlement but “the prioritization of sport over other urgencies which characterize a new social context may also interfere with the establishment of a sustainable daily routine and slow down the socialization in other social contexts.” This finding resonates with ethnographic studies of the sport participation of refugee-background young men who may be “seduced” by the appeal of an elite sporting career at the expense of more likely pathways for social mobility, notably education and employment (Spaaij, 2012).

### Methodologies and Ethics

**Figure 3:**
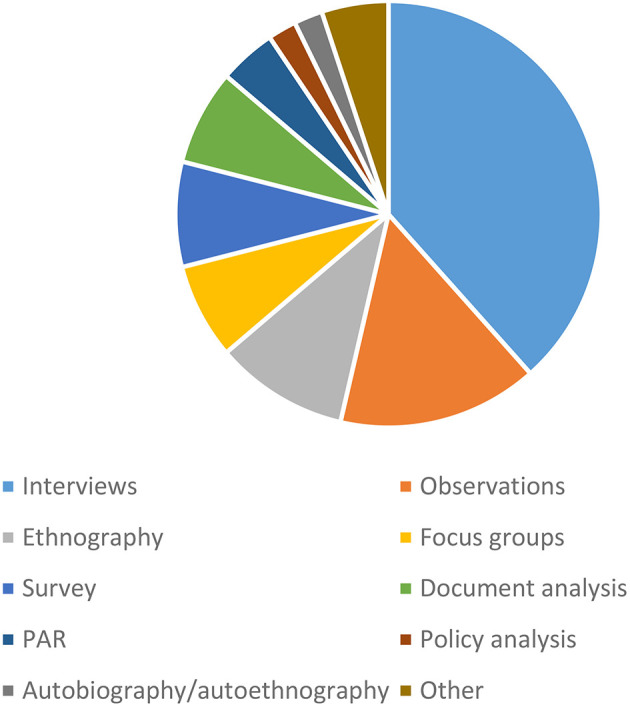
Research methods used.

With regard to methodology, the vast majority of studies were qualitative (*n* = 64, or 76%). Four studies were exclusively quantitative, and eight studies employed a mixed methods design. Figure 3 summarizes the main qualitative and quantitative research methods used in the literature. Among the qualitative publications, interviews (ethnographic), observations, focus groups, and document analysis (secondary data) were the most commonly used methods of data collection (classification adapted from Johnson and Turner, 2003). Data collection methods, such as participatory action research, autobiography/autoethnography, photo elicitation, reflective journaling, drawings, and film analysis were used to a much lesser extent. The quantitative studies typically used self-report questionnaires. Only one study used a randomized control trial (Nordbrandt et al., 2015). Mixed methods research combined some of the qualitative and quantitative methods; this typically involved a combination of questionnaires and interviews or focus groups.

A noteworthy feature of methodological aspects of the literature is the limited discussion afforded to ethical challenges and considerations in research with refugees and forced migrants. This is problematic because ethics is both vital and complex in this field of research, and it has long been a prominent area of debate in refugee and forced migration studies (e.g., Mackenzie et al., 2007; Block et al., 2012). The studies included in this review typically limit their discussion on research ethics to brief statements regarding institutional ethics approvals or how participants' informed consent was obtained. For instance, Ley et al. (2017) note that participants were informed about their right to withdraw at any time without negative consequences, for example toward their asylum process or therapy. The latter is an important consideration given research participants' potentially precarious legal and health status. Only a few studies provide more detailed reflections on ethical challenges and the strategies used to mitigate these, for example with regard to co-creation (Robinson et al., 2019), field roles (Spaaij, 2013; Mohammadi, 2019), and reflexivity and researcher positionality (Dukic et al., 2017; Abur, 2018; Fader, 2018). Doidge (2018) offers valuable critical reflections on the politics and ethics of research and activism in this field. He rightly observes that working in this context requires emotional labor and critical engagement, including ongoing recognition and action toward the power dynamics of working with vulnerable groups. Furthermore, Blanchard (2018) discusses the use of oral consent as a more suitable and culturally appropriate option compared to traditional written consent when working with refugees and asylum seekers with low levels of linguistic or literacy comprehension. We will return to the issue of research ethics later in this paper. First, we turn to the key themes, issues, and challenges contained within the literature.

## Research Themes

The main themes in the literature are 3-fold: health promotion, integration and social inclusion, and barriers and facilitators to participation. There is considerable overlap between these themes in the research surveyed, especially in studies that examine both barriers to participation and the outcomes of participation/intervention. Overall, the thematic foci of research fall largely along disciplinary lines. Distinct groupings can be identified: (1) studies situated in the health sciences, which focus primarily on health problems and health promotion (i.e., sport and physical activity as medicine); and (2) social science research, which typically focuses on questions of integration/inclusion and barriers to participation. Below we discuss each theme in more depth.

### Health Promotion

Health promotion concerns the process of enabling people to increase control over determinants of health and thereby improve their health. The literature on sport, physical activity, and forced migration echoes this definition. In this literature, forced migration and resettlement are associated with distressing circumstances that contribute to poor physical and psychological wellbeing including high rates of trauma and other stress-related disorders (Hashimoto-Govindasamy and Rose, 2011; Ley et al., 2018; Ley and Rato Barrio, 2019). Physical activity among refugees and forced migrants is frequently framed through the lens of mental and physical health outcomes, such as fitness, general wellbeing, self-efficacy, and reduced stress. It has also been argued that participation in physical activity interventions can provide a pathway to structure, routine and re-gaining control (Hartley et al., 2017). Widely discussed in the literature are positive health outcomes connected to social benefits, such as (inter)cultural understanding, social connectedness, and the potential of establishing an entry point to structured sport participation (Olliff, 2008; Nathan et al., 2010, 2013; Bunde-Birouste, 2013). It is at this juncture that the lens of health and wellbeing intersects with the theme of integration and social inclusion.

### Integration and Social Inclusion

The *Home Office Indicators of Integration framework 2019* recognizes leisure as a marker and means of integration. Leisure activities can help newly arrived migrants “learn more about the culture of a country or local area, and can provide opportunities to establish social connections, practice language skills and improve overall individual health and wellbeing” (Ndofor-Tah et al., 2019: p. 38). This assumption is strongly reflected in the scientific literature. Already in 2012, Spaaij called for sport and leisure to be considered a marker and means of refugee integration. Sport activities can function as a supportive environment where information regarding other services and systems can be shared and where trusting relationships with coaches and youth workers can be developed (Olliff, 2008). Furthermore, sport participation is described as a process to facilitate acculturation, integration, inclusion, and feelings of belonging, as well as to connect refugee-background young people to other people with shared experiences and to people who share a similar cultural background (Nathan et al., 2013; Spaaij, 2015; Baker-Lewton et al., 2017; Seiberth et al., 2018). A sense of belonging or community can be fostered through participation in a sports team (Nathan et al., 2013; Whitley et al., 2016; Fader, 2018; Stone, 2018) or in co-ethnic diaspora events (Spaaij and Broerse, 2019). Hurly (2019) and Hertting and Karlefors (2013) describe how different cultural meanings are attached to sport, which suggests the diversity of potential outcomes of sport participation. In other words, there is no “one-size-fits-all” approach to understanding the relationship between sport and integration.

What is evident, however, is that relatively few studies take a critical approach to integration. To varying extents, research tends to deploy constructions of integration that are aligned with those of assimilation, whereby sports activities are considered a context or means for refugees and forced migrants to learn and embody dominant cultural norms. Some studies, such as Henry (2009) and Jeanes et al. (2015), explicitly critique this tendency in relation to current sport policy and practice. The assimilationist tendencies within sports institutions and research are not unique to this particular field of research; they have been plaguing the wider field of migration and sport for decades (Alkemeyer and Bröskamp, 1996; Smith et al., 2019).

### Barriers and Facilitators to Participation

Refugees and forced migrants tend to experience barriers to participation in sport and physical activity, especially in relation to structured, club-based sport. The literature demonstrates that barriers operate at multiple levels: structural, sociocultural, interpersonal, and personal (Spaaij, 2013; Baker-Lewton et al., 2016). Structural barriers include policy gaps, transport constraints, lack of funding, resource constraints, and the lack of communication and collaboration between sporting and supporting organizations. Lack of (or inequities in) government policy and investment, particularly a lack of welfare support, acts as a powerful barrier to sport and physical activity for refugees and forced migrants (Hartley et al., 2017). Moreover, Burrmann et al. (2018) highlight organizational culture and the human resources available in sports clubs as important factors that influence access and participation. Sociocultural barriers to participation may include lack of inclusive sporting practices, social or community norms that constrain participation opportunities (e.g., for girls and women), and the absence of family or parental support (Olliff, 2008; Spaaij, 2013; Abur, 2018). Personal constraints include language barriers, lack of time or family responsibilities, limited knowledge about sport systems or participation opportunities in the destination country, and the absence of pre-existing sport participation experience or skills (Hancock et al., 2009; Spaaij, 2013; Mohammadi, 2019). Mohammadi (2019) illustrates the complexity of personal constraints. The adult female refugees and asylum seekers in her research had not had the chance to learn how to ride a bicycle in their homelands due to societal restrictions imposed on women. She found that “the preconception of women cycling in public and the fear of stigma associated with it as well as the lack of socialization agents negatively influenced the women's acquisition of cycling skills” (Mohammadi, 2019: p. 1090). After perceiving the cultural value and pleasures associated with cycling in their country of resettlement, Germany, they were motivated to develop cycling skills. Yet, their limited knowledge of the German language prevented them from building a strong connection to other female asylum seekers and local residents through cycling.

In contrast to the aforementioned publications, which primarily describe barriers that participants experience, Anderson et al. (2019) report on the perceptions of sport service deliverers/managers and the challenges they face in delivering suitable sports activities for refugees. The authors conceptualize societal, organizational, interpersonal, and intrapersonal factors in a socio-ecological model to highlight how each level influences the design, delivery, and outcomes of sport for health programs. Spaaij (2013) uses a similar socio-ecological model, but instead foregrounds the lived experiences of participants.

A major factor that can act as a barrier to participation in sport and physical activity is discrimination, which operates at all of the aforementioned levels. Even though sport participation may contribute to integration and social inclusion outcomes, it can also have the opposite effect of exposing participants to racial or religious discrimination. Baker-Lewton et al. (2017) and Abur (2018) detail South Sudanese Australians' experiences of everyday racism in sport and demonstrate its impact on experienced exclusion, while Spaaij (2012, 2013) documents similar experiences among Somali Australians. Whereas, Baker-Lewton et al. (2017: p. 12) describe racism as “a historical and ongoing social force that poses serious challenges to the belonging of young people of refugee background,” Ravaglia (2018) describes how racism is challenged through the creation of an inclusive club culture and symbols, such as the use of the banner “Refugees Welcome” (see also Trigo and Freitas, 2017). In the Croatian context, Jurkovic (2019) describes the complexity of inclusive environments that might be supported by some members but opposed by others who view “left-wing activism” as a distraction to football training sessions and matches.

The aim of identifying barriers to participation is to create awareness and, ultimately, overcome barriers. However, the barriers model is problematic where it exhibits implicit assimilationist tendencies, by illustrating how refugees and asylum seekers could be incorporated into the mainstream, or where takes a particular negative view of multiculturalism, one that interprets ethnic and cultural differences as barriers to participation (Donnelly and Nakamura, 2006). In this context, O'Driscoll (2016) demonstrates that Western practices and mainstream perceptions are not always appropriate, and that researchers need to be considerate of Western theory being applied to non-Western population groups, in the case of her research, the Karen community in Melbourne, Australia.

It is also important for research to do justice to the heterogeneity of refugee and forced migrants. The relevance of particular barriers differs according to, *inter alia*, age, gender, social class, and time in a new country. Even within particular groups, there are important differences that affect individuals' sports experiences. For example, Spaaij (2013) found that second-generation Somali Australians, and particularly young males, tended to have a much stronger interest and time investment in sport participation compared to the first generation, whose priorities were work, family, housing, employment, and physiological needs. Compared to men, female respondents were less likely to feel they had the freedom and opportunity to participate in sport and physical activity. Abur (2018) observed similar differences. Of particular note in this regard is the existence of a body of the literature devoted to refugee women's settlement through sport and physical activity. For refugee women, additional barriers include norms and expectations within their country of origin and their ethno-cultural communities (Guerin et al., 2003; Palmer, 2009; Pizzolati and Sterchele, 2016; Mohammadi, 2019). According to the literature, supporting refugee women in their settlement journeys can be pursued through community development approaches that emphasize group interests, cultural norms, and strengths. These approaches can facilitate refugee women's engagement and access to services (Guerin et al., 2003; Hashimoto-Govindasamy and Rose, 2011).

Most studies that examine barriers to participation provide at least some practical recommendations for reducing such constraints. A few studies, however, analyze opportunities and facilitators to participation in greater depth. The aforementioned studies by O'Driscoll (2016); Mohammadi (2019), and Robinson et al. (2019) are cases in point. Collectively, these studies discuss, for instance, the need to understand the target group's culture, needs, and context, importance of low financial cost, geographic proximity, and flexible delivery, the need to transfer decision-making power in programming to the target group, engagement of families, building facilitators' intercultural competences, and gender-segregated provision.

### The Role of Sport and Physical Activity

Within the three identified thematic foci, sport and physical activity appear in a variety of ways. The studies included in this review can be grouped into two main categories with regard to the centrality of sport and physical activity within the analyses presented. The first group of studies (*n* = 77) has sport, physical activity, or leisure among refugees and forced migrants as a core analytical focus. These publications represent the current canon of scientific research in this field of studies. A second, much smaller group comprises refugee and forced migration studies (*n* = 6) that did not focus specifically on sport and physical activity but on refugee and forced migrant wellbeing and settlement more broadly. These studies nonetheless yielded relevant findings, even if they were peripheral to the main thrust of the studies. For example, Apriadi and Juliantoro (2018) identify access to sport and recreation as a fundamental right for refugees and asylum seekers in immigration detention. They consider the fulfillment of the right to physical activity and recreation as one of seven indicators of human rights protection in an Indonesian detention facility. Uptin's et al. (2013) study of African-background former refugees' experiences in Australian high schools delivers important insights for the study of sport and forced migration. They concluded that there was limited scope for the young former refugees to develop non-racially stereotyped youth identities within the school setting. Sport was one of the few spaces that was available to them, and particularly to boys, to perform the acceptable racialized identity of “cool black basketball player” (Uptin et al., 2013: p. 132), from which they derived social status and relationships across class and ethnicity. By contrast, girls did not perceive sport as a way to be included in their school friendships; consequently, they had to find other ways to be included in social groups, notably through music. These findings shed important light on the limitations of sport as a vehicle for social advancement forrefugee-background youth.

## Fundamental Concepts, Issues, and Problems

Our synthesis of the literature reveals a number of recurring concepts, issues, and problems. In this section, we consider three key aspects: categorization, deficit approaches, and intersectionalities.

### Analytical and Policy Categories

The definitions and categories used to describe the objects and subjects of research matter because they shape how we formulate research questions and where we focus our analytical attention. The terms used to refer to research participants is highly diverse. The most commonly used modes of identification for research participants were “refugees” and “people with refugee backgrounds.” Other popular modes of identification included “asylum seekers” and the broad term “newly arrived migrants.” Studies also referred to participants as “migrants,” “forced migrants,” “people seeking asylum,” “war and torture survivors,” “former refugees,” “boat people,” “illegal immigrants” or “diaspora.” The usage of these terms was frequently problematic in that it conflated different terms, such as “refugee” and “asylum seeker,” which may render invisible certain important differences, such as the more restricted legal and labor rights of asylum seekers vis-à-vis those with official refugee status (Amara et al., 2004; Stone, 2018). Stura's (2019) conflated use of the terms “refugee” and “asylum seeker” is a case in point. Interviewees are referred to as refugees but the article reveals that 91 per cent are in the process of seeking asylum, and hence not officially recognized as refugees. In the reviewed literature, categories were often applied loosely to refer to people at various stages of migration and settlement. The term “refugee” may refer to: (1) migrants who have arrived in the new country and have been assigned official refugee status; (2) migrants in transit, fleeing persecution; (3) asylum seekers waiting for their case to be processed and residing in refugee camp or elsewhere in home, host or third country; (4) former refugees (Spaaij et al., 2019). The latter category refers to people who have lived in a new country for some time and may have permanent residency or citizenship status in that country. The risk here is that, in the absence of more precise language, people who have experienced humanitarian migration at some point in their life are potentially lumped into a single “refugee” category for research purposes.

In some studies, more stringent categories and distinctions were employed. This is particularly the case for the 20 studies that deployed official UNHCR (*n* = 17) or national government (*n* = 3) definitions of refugees and asylum seekers as their initial frame of reference. While this use of official legal and policy definitions helped to prevent some of the aforementioned conceptual conflation, it raised another problem that is well documented in refugee and forced migration studies: an over-reliance on policy categories. Bakewell (2008) argues that forced migration research tends to be framed around policy categories and priorities, often in an attempt to ensure that the findings are relevant and serve to improve the circumstances associated with forced migration. However, this reliance on policy categories “limits academic research by constraining the type of questions asked, the objects of study and the methods and analysis adopted” (Bakewell, 2008: p. 433). Only three studies demonstrated an explicit awareness of the need to make the assumptions and categorizations of policy makers visible and open to inspection. While Woodhouse and Conricode (2017: p. 941) acknowledge that the term “asylum seeker” is used in legal and popular discourse, they instead used the phrase “people seeking asylum” in response to “participants' attempts to articulate identities on their own terms and their highlighting of the limiting nature and negative perceptions of the term ‘asylum seeker'.” Dukic et al. (2017) use the term “people seeking asylum” for the same reason. In a similar vein, Amara et al. (2004) categorize refugees as newly established minorities. They note that “some refugee communities may refuse to be marked in public as refugees since they wish to be completely, culturally or politically, assimilated as British ethnic minorities” (Amara et al., 2004: p. 81). Others, such as Spaaij (2013) and Spaaij and Broerse (2019), also use participants' self-identifications as a frame of reference. In these studies, there is a recognition of the potential injustice associated with labeling participants in ways that they deem stigmatizing or that do not reflect the complexity and temporality of the social issues and transitions they navigate.

Critical consideration of the relationship between policy categories and analytical (research) categories is vitally important. Bakewell (2008: p. 438) points out that if research uncritically accepts the boundaries of the field imposed by policy categories, it will tend to confirm and legitimize the assumptions made by powerful actors, such as states, and ensure that these assumptions remain taken-for-granted. Northcote and Casimiro's (2009) critical analysis of evidence-based resettlement policy in Australia exemplifies this vital point. Their study of a sports program for Muslim refugee youth demonstrates a disconnect between government discourse, which prioritizes participation in structured, organized sport, and the young people's needs and aspirations, which focus on *ad hoc* sports events and self-organized sport participation. This study indicates that when researchers are uncritical in their approach, they run the risk of making their research an instrument for social control by state or regulatory discourses. Sports program deliverers play an equally important role in the reproduction of regulatory discourses. In her study of a sport-based settlement program for newly arrived refugees and other migrants, Broerse (2019: p. 245) found that “while clients were talked about in respectful and empowering terms in meetings or informal discussions, policy documents […] and consent/evaluation forms reinforce migrant categorization.” The focus on participants' migratory, non-Australian, background and the visa on which they entered the country frames participants in a reductionist way (Broerse, 2019). This leads us to a related issue in the literature: deficit vs. strengths-based approaches.

### Deficit vs. Strengths-Based Approaches

Much of the literature exhibits a deficit-based paradigm that associates refugee status with very depressing circumstances, such as trauma, poor health, deprivation, and social isolation (Spaaij and Oxford, 2018). For example, Stura (2019: p. 1) emphasizes that refugees have experienced “major trauma” and “come from countries with significant cultural differences” that make their acculturation in the destination country difficult. Noteworthy in this study is that whereas volunteers stressed heavy baggage and negative experiences, refugees themselves did not talk about these experiences. In a similar vein, Renzaho et al. (2012) frame African refugees and migrants in terms of increased risk of obesity and obesity-related diseases, while Hartley et al. (2017) and Mohammadi (2019) emphasize the impact of experiences of war, displacement, and family separation on health, wellbeing, and social exclusion.

While the challenges described in the studies are real and meaningful, the discursive linkage of the category of “refugee” to such depressing circumstances contains the risk that we fail to see the “normality” and agency of forced migrants (Bakewell, 2008), and that research reproduces stereotypes (Spaaij and Oxford, 2018). This concern is addressed in seven publications (10% of the total) that reference the need for a strengths-based approach that recognizes forced migrants' strengths, capabilities, knowledge, and resources. Evers (2010: p. 61) argues that “sport-based intervention programs being mobilized to “fix” [refugee-background] young people is a misplaced agenda.” He highlights “an obligation to become intimate with the young people's perspectives to the point of finding out not what the young people “need” to know but what these young people's skills at life, settlement and well-being may teach *us* as researchers, as sports facilitators, as youth workers, as community development officers, volunteers, and the like” (Evers, 2010: p. 57; emphasis in original). In a similar vein, Baker-Lewton et al. (2017) challenge dominant political and media representations of young South Sudanese men as both “violent” and “traumatized.” Other studies point to the internal diversity of refugee “groups,” noting that despite commonalities, there are important differences in experience among refugee and asylum seekers (Spaaij, 2013; Abur, 2018). Uptin et al. (2013: p. 135) provide an important critique of the deficit model by concluding that:

schools that view “the refugee” as a hegemonic [*sic*] group overlook the individual's needs and strengths of former refugee students and lose sight of their diverse backgrounds. This positioning, as seen in this research, keeps former refugee students in a deficit position and confined to lower classes.

On the occasions that schools nurtured the young people's talents and opportunities, these were largely restricted to aforementioned stereotyped positions, such as competent athletes.

### Intersectionalities

Another problem emanating from a deficit-based paradigm is that it tends to assume that their situation is associated with their refugee status. Bakewell (2008: p. 445) notes a

danger of falling into the trap of assuming that a certain set of problems or experiences are the exclusive domain of refugees. This can too easily lead us to ascribe particular problems to a person's identity as a refugee, when it may be more closely related to other aspects of their identity which might be shared with other “non-refugees” in the local population: membership of an ethnic group, length of residence, income level, level of education, and so forth. The challenge is to identify where refugee identity may be salient in creating a different set of social interactions.

This critique applies to the majority of studies in this review, whose analyses tend to emphasize research participants' (policy-ascribed) identity as refugees as a salient, if not the most salient, independent variable. An important exception is Hertting and Karlefors's (2013) study of the sports experiences of newly arrived migrant children in Sweden, which is sensitive to similarities between the experiences of forced migrant and non-migrant children. They reason that the themes that emerged from their study

would also appear in a similar study of children with a native Swedish background. There are also native Swedish children who are spectators and/or who are not involved either in spontaneous sports or in club sports. Some Swedish-born children prefer spontaneous sports rather than club sports. The reasons for this can vary, and are not necessarily cultural or religious, but can be social or economic. It may be a matter of bodily ideals, talent, or economic conditions, or simply lack of interest (Hertting and Karlefors, 2013: p. 42).

The authors' reference to social and economic factors suggests the need for intersectional approaches to the study of sport and forced migration (Spaaij et al., 2019). Intersectionality must be used carefully, highlighting the multidimensionality of identity rather than being employed as a categorical addition. It acknowledges the fact that refugees and forced migrants are an extremely diverse group of individuals with unique journeys and experiences, as noted explicitly in four publications (Amara et al., 2004; Stack and Iwasaki, 2009; Spaaij, 2012, 2013). Furthermore, their experiences of resettlement and integration vary greatly depending on resource allocation, support systems, personal and community resilience, and community attitudes and perceptions in the destination country. Identifiers (such as age, ability, socioeconomic status, religion, gender, and sexuality) and their intersections may affect settlement processes as well as access to or interest in sport and physical activity. Taking an intersectional approach in research is critical if we are to avoid the trap of unreflexively ascribing particular problems or experiences exclusively to a person's identity as a refugee. Despite some initial efforts (e.g., Blanchard, 2018), a fully-fledged intersectional approach is yet to be developed in this field of research.

## Research Gaps

In the preceding sections, we have discussed the main characteristics, themes, issues, and challenges of the scientific literature on sport and forced migration. The identified problems regarding policy categories, deficit approaches, and intersectionalities constitute important opportunities for future research. In this section, we build on these challenges to formulate four research gaps that warrant greater attention in this field of research. These gaps concern experiential dimensions of sport and physical activity, the need to decolonize research, the space for innovative methodologies, and research ethics.

### Experiential Dimensions of Sport and Physical Activity

The dominant themes in the literature reveal an instrumentalist tendency in research in that it tends to align itself with two current policy priorities: (1) sport as a means for promoting the integration and wellbeing of refugees in destination countries; and (2) increasing the sport participation of under-represented population groups. Both policy and research primarily frame sport as a means to an end, that is, “sport for inclusion” and “sport as medicine.” This is reflected in the theories and concepts that underpin the studies included in this review. The dominant theoretical frameworks in this field of research have been that of social capital (*n* = 9), belonging (*n* = 5), acculturation (*n* = 4), and social ecology (*n* = 4; in studies of barriers and facilitators). Other theoretical frameworks deployed in the literature are (postcolonial) feminism (*n* = 3), integration (*n* = 2), community development (*n* = 2), and liminality (*n* = 2), among others. What the dominant theories have in common, with the exception of some applications of belonging theory, is that they privilege cognitive perceptions over refugees' and forced migrants' emotional and bodily experiences of sport and physical activity. We would argue that these sensory experiences are essential if we are to truly understand the meaning of sport and physical activity in the everyday lives of refugees and forced migrants.

Only two studies paid central attention to the body and/or emotions as core components of behavior and experience. Evers (2010) offers a vital exploration of the role of intimacy and affective connections on and off the sporting field, which he believes is essential in understanding how young refugee men experience sport programs and the migration-related challenges they face. Ley et al. (2017) also provide some insight into bodily and affective experiences, but largely from a psychological and trauma-focused perspective. Their study of flow in sport and exercise therapy with war and torture survivors reports how, for some participants, sport and exercise facilitated the experience of pleasure, distraction and respite from illness-related thoughts and worries, and being in the present in ways that were therapeutically meaningful for this population. We conclude that there is a need for more research into emotional and bodily sensations and dynamics of sport and physical activity among refugees and forced migrants, for which Evers (2010) in particular offers an important starting point.

### Decolonizing Research

Our analysis of the geographical distribution and thematic focus of the academic literature reveals a Eurocentric bias. There is a stark discrepancy between the research sites of the published studies and the locations where the majority of the world's displaced people actually reside. One explanation is the location of universities and their research capacities within (re)settlement countries. Another explanation for this discrepancy is the aforementioned tendency in research to align itself with the policy priority of sport as a means for promoting the integration and social inclusion of refugees in destination countries. It could be argued that this research prioritization of integration monitoring is a neocolonial form of knowledge production (Schinkel, 2018). It can be argued that only by breaking away from a narrow focus on this policy priority it will be possible to challenge some of the Eurocentric bias in the literature. At a deeper level, however, it will require the decolonization of research.

The first and foremost proposition of decolonial research is the need to move beyond Eurocentric paradigms. This is important as research “is one of the ways in which the underlying code of imperialism and colonialism is both regulated and realized. It is regulated through the formal rules of individual scholarly disciplines and scientific paradigms” (Tuhiwai Smith, 1999: p. 7). Unequal power relations between researcher-researched were at the core of disciplines, such as anthropology when these were established. Unequal relations and other ways of carrying out research continue to define Western research paradigms that can be dismantled by following a decolonial research approach. Although the decolonialization movement is rooted in Indigenous rights movement, Land (2015: p. 249) writes that it can be applied to refugee studies as the “key dilemmas negotiated by social justice supporters concerned with supporting the agendas of marginalized groups and polities other than Indigenous peoples.” Besides supporting agendas of the marginalized (and oppressed) and offering avenues for different ways of knowing, decolonial methods foreground the principles of cultural protocols, ethics, co-design, and dissemination of research findings (Tuhiwai Smith, 1999). This approach is yet to be seriously explored in this field of research. For example, if we consider authorship of the published research, it is apparent that it is still overwhelmingly white European, North American and Australian (male and female) scholars writing about the experiences and voices of “the others.”

### Innovative Methods

Earlier in this paper, and related to the decolonization of research, we established that the vast majority of studies have used relatively traditional research methods, and that engagement with alternative methods is still in its infancy. Exceptions are the emergent interest in participatory action research and the (slow) adoption of basic forms of visual methodology, such as drawings, photovoice, and film analysis. Other methods, which have been gaining traction in both forced migration studies and sports studies, such as autoethnography and institutional ethnography, are still unexplored in studies of the intersection between sport and forced migration.

We see three promising methodological directions for this field of research. First, there is an opportunity to complement single (qualitative) case studies, which constitute the bulk of existing research in this area, with robust mixed methods designs that bring together meaning and measurement by integrating qualitative and quantitative approaches. Only 10% of publications were based on mixed methods research. Second, and related to the first point, there is a complete absence of longitudinal research in the literature. None of the 83 publications employed a longitudinal research design. Longitudinal research provides information about changes over time that are highly relevant to understanding experiences and outcomes of sport and physical activity. Some of the ethnographies conducted in this field of research also capture changes over time (e.g., Spaaij, 2015; Ravaglia, 2018), but the literature is yet to engage fully with longitudinal studies. We recommend that scholars in this field of study consider how longitudinal designs can be applied effectively in their work, and how methodological and ethical areas of learning can be addressed, for example with regard to retention of participants over time and the adaptation of research tools to changing contexts and life stages (McMichael et al., 2014).

Third, there is space to progress transnational methodologies to counteract the dominance of methodological nationalism in the literature. Methodological nationalism is a mode of thinking and knowledge production in which the nation-state is seen as natural basis of analysis; in the literature, this is most visible in studies of sport as a means for integration in a destination country (e.g., Australia, United States, Canada, Germany). In forced migration studies (Marlowe, 2017) as well as in sports migration studies (Carter, 2011; Agergaard, 2018), there has been a growing emphasis on transnational mobilities, flows, and connectedness as key foci of analysis. This emphasis exists alongside an acknowledgment that national identity and sovereignty are still “a powerful signifier that continues to make sense for different actors with different purposes and political implications” (Wimmer and Glick Schiller, 2002: p. 326–327). This transnational orientation is yet to fully penetrate the field of sport and forced migration research. Only two published studies adopt what can be called a transnational research approach (Michelini, 2018; Spaaij and Broerse, 2019).

### Ethical Relationships in Research

Critical reflection on the challenge of constructing ethical relationships in this field of research is required. As noted, our findings indicate that limited discussion is afforded to ethical challenges and considerations within the current literature. This is in stark contrast to the prominent role that such discussions have played in refugee and forced migration studies. We strongly encourage scholars to engage more actively in this discussion and to address key lessons and strategies within their research.

The studies included in this review tend to embody standard interpretations of informed consent, which are often inadequate or inappropriate in the context of refugee research. Researchers typically obtained informed consent through the one-off provision of written or verbal information statements or by asking participants to sign a consent form. In contrast, refugee and forced migration scholars have proposed an iterative model of the consent process, whereby consent is an ongoing negotiation (Mackenzie et al., 2007). Iterative consent establishes the research as a partnership, enabling refugee participants to play a more active role in setting the research agenda so that it is responsive to their needs and respects their concerns and values. It can also assist in building and sustaining trust, including the consideration of how the research is shared, disseminated, and used. At a deeper level, research with refugees and forced migrants entails an obligation to ensure that the research provides reciprocal benefits for those concerned, whether in the form of developing skills and capacities, improving health and social outcomes, influencing policy, or enhancing the quality of programming (Mackenzie et al., 2007; Block et al., 2012). Yet, the current literature on sport and forced migration rarely addresses the issue of reciprocal benefits explicitly and robustly. Herein lies a vitally important issue for future research and debate. In this regard, lessons can be learned from the handful of studies that adopted participatory research approaches (Stone, 2013; Robinson et al., 2019) and critically reflexive approaches (Doidge, 2018).

## Conclusion

In this paper, we have critically synthesized what is known about the sport and physical activity experiences of people with refugee and forced migrant backgrounds, and identified key issues and directions for future research. The analysis shows an increase in the volume of published research on the topic in recent years, concentrated primarily in Western countries around the themes of health promotion, integration and social inclusion, and barriers and facilitators to participation in sport and physical activity. The review of literature highlights categorization, deficit approaches, and intersectionalities as three pressing challenges in this area of research. Based on this synthesis, we identified four research gaps that require greater attention in future research: experiential dimensions of sport and physical activity, the need to decolonize research, the shortage of innovative methodologies, and research ethics.

There are limitations associated with any type of literature review, and this review is no exception. A limitation of the review is that it does not fully capture the fluid and evolving boundaries of this area of study vis-à-vis migration studies more broadly. As noted earlier, it is likely that some research in the broader area of “sport and migration” covers aspects of refugee and forced migration experience (e.g., within their sample of participants) without explicitly referring to the categories of “refugee,” “forced migrant,” or “asylum seeker.” The literature search process and the associated inclusion criteria are unlikely to have identified all of these studies exhaustively. Herein, too, lies a lesson for critical reflexivity regarding potential disjunctures between the analytical and policy categories we adopt in our research. Another limitation concerns the inclusive nature of the review, especially the inclusion of dissertations and book chapters that may not have been subjected to rigorous, double-blind peer review in the same way that scientific journal articles are. Additional quality assessment of the methodologies and theoretical applications of these studies beyond what is offered in this review would be beneficial.

A strength of this literature review is the inclusion of several foreign languages in order to raise awareness of, and obtain insights from, relevant research published in other languages. The inclusion of scholarly books, book chapters, and dissertations is another unique feature of this review. While this approach directly addresses the identified need to decolonize research methodologies, we acknowledge that our study most likely does not cover the full range of foreign-language research conducted in diverse world regions, such as the Middle East, Asia, and Africa. We recognize that this research constitutes equally legitimate knowledge that should inform global scholarship and debate. We encourage researchers to engage with the full breadth of studies in other languages, societies, and publication outlets that can inform our understanding of the intersections of forced migration, refugee settlement, and sport and physical activity across the world.

## Author Contributions

This publication was produced by the Sport, Diversity and Social Change Research Group at Victoria University as part of its We Play: Refugee Settlement through Sport project. All authors have made a substantial, direct and intellectual contribution to the work, and approved it for publication. RS, JBr, SO, CL, and BK were involved in the design of the study and contributed to the review of literature. RS wrote the first draft of the manuscript, after which JBr, SO, CL, FM, BM, BK, LL, AP, and JBi read and contributed to the revision of the manuscript.

### Conflict of Interest

The authors declare that the research was conducted in the absence of any commercial or financial relationships that could be construed as a potential conflict of interest.
